# Medication use and fall risk among older adults in long-term care facilities: A cross-sectional analysis

**DOI:** 10.17159/2078-516X/2025/v37i1a20605

**Published:** 2025-08-15

**Authors:** N Ebrahim, J Ras, R November, L Leach

**Affiliations:** Department of Sport, Recreation and Exercise Science, Faculty of Community and Health Sciences, University of the Western Cape, Robert Sobukwe Rd, Bellville, Cape Town 7535, South Africa

**Keywords:** retirement homes, polypharmacy, elderly

## Abstract

**Background:**

Falls among older adults are a major concern, frequently resulting in injuries and a decreased quality of life. While medication use is known to be a key contributor to fall risk, the specific medications have not been thoroughly investigated. This study is vital to investigate the relationship between specific medications and fall risk, aiming to strengthen safety measures and minimise falls among the elderly in care facilities.

**Objectives:**

To determine the relationship between medication use and the risk of falling among the elderly living in Long Term Care (LTC) facilities.

**Methods:**

The study used a cross-sectional design to investigate males and females, aged 60 years and older, living in retirement facilities in the City of Cape Town. A convenient sampling method was utilised to recruit a total of 258 participants from multiple long-term care facilities in Cape Town, South Africa. The Spearman’s rank correlation coefficient was used to determine associations between falls, fall risk factors, and various medications used.

**Results:**

Majority of participants were at low risk (81%; n=211), 15.1% (n=39) at moderate risk and 3.1% (n=8) at high risk for falling. Antidepressant [χ^2^ (1)=4.941; p=0.026; OR=2.083 (95% CI: 1.082, 4.012)] and anti-diabetic [χ^2^ (1)=4.097, p=0.043, OR=2.070 (95% CI: 1.013, 4.228)] medications were the only drugs significantly associated with falling.

**Conclusion:**

These findings highlight the significant association between antidepressant and anti-diabetic medications and an increased risk of falls. Effective medication management and fall prevention strategies are essential among this vulnerable population. Clinicians should carefully assess the risks of these medications when prescribing to older adults and consider alternative treatments when necessary.

Falls among older adults are multifactorial, with medication use being one of the key contributing factors. Comprehensive fall prevention programs in long-term care (LTC) facilities typically adopt a multifaceted approach that addresses medication management, environmental modifications, exercise, and staff education.^[1]^ Many elderly residents in LTC facilities experience multiple chronic illnesses, along with declining physical and mental functioning, which significantly increases their likelihood of falling.^[1]^ As people age, the progressive decline in physical and cognitive abilities often makes it difficult to live independently within the community, leading many older adults to move into LTC facilities. Unfortunately, this transition tends to exacerbate their risk for poor health outcomes and imposes an additional financial burden.^[2]^

Various individual factors, such as age, comorbidities, cognitive function, and physical impairment, influence the relationship between medication use and fall risk. Older adults with multiple chronic conditions and frailty are especially vulnerable to the adverse effects of medications.^[2,3]^ Research has consistently shown that the use of multiple medications increases the risk of falls, often due to medication interactions, as well as changes in cognitive and physical function, all of which contribute to a heightened fall risk.^[1]^

Several studies^[1–3]^ have identified specific classes of medications as particularly associated with increased fall risk among older adults. These include antidepressants, benzodiazepines, and anti-psychotics, which are associated with sedation, dizziness, and impaired balance—factors that increase the likelihood of falls. To address these risks, interventions often involve reviewing and managing medications. These strategies may include discontinuing unnecessary medications, adjusting dosages, and exploring alternative treatments to minimise adverse drug events and reduce fall risk.^[4]^

While all individuals are susceptible to falls, the elderly population is particularly at risk due to ageing-related physiological changes and various health conditions.^[5]^ These age-related changes, combined with cognitive impairments, medication use, and environmental hazards, significantly increase the likelihood of falling and impair the ability to maintain balance.^[6]^ The consequences of falls among older adults can be far-reaching, including physical, social, psychological, and financial impacts.^[7]^ In hospitals, over 84% of incidents reported were related to falls, often leading to longer recovery times and more complicated rehabilitation.^[8]^

Therefore, this study aims to explore how specific medications, such as antidepressants and anti-diabetic drugs, influence fall risk among older adults, specifically in long-term care facilities and examine the implications of these findings for medication management practices in these settings.

## Methods

This study employed a quantitative cross-sectional design to explore the relationship between medication use and fall risk among older adults living in long-term care facilities. A convenient sampling method was utilised to recruit a total of 258 participants from multiple long-term care facilities in Cape Town, South Africa. The study included residents aged 60 years and older living in long-term care facilities. Informed consent was obtained from all participants. A researcher-generated questionnaire was used to collect data on participants’ demographic information, medical history, and medication use. Medications were categorised into the following groups: anti-hypertensives, anti-epileptics, anti-psychotics, diuretics, anti-Parkinson’s medications, antidepressants, sedatives, insulin and diabetic medications, and other medications not included in the categories as mentioned above. The Fall Risk Assessment Tool (FRAT) was a previously validated 4-item fall risk assessment tool that also collected information on medication use (not taking any, taking one, taking two, or taking more than two). The Timed Up-and-Go (TUG) test was used to assess fall risk and the progression of balance. The Berg Balance Scale (BBS) is a 14-item scale used to evaluate static balance objectively. The Dynamic Gait Index (DGI) assessed eight components of gait and was used to determine the functional balance. Ethical approval was obtained from Biomedical Research Ethics Committee (BMREC) at the University of the Western Cape.. Informed consent was obtained from all participants before data collection. Participant confidentiality and privacy were maintained throughout the study process.

### Data analysis

Descriptive statistics, including frequencies, percentages, means, and standard deviations, were used. Pearson’s Chi-square test was employed to explore associations between medication use and fall risk. Polypharmacy was defined in this study as the use of three or more medications. Statistical Package for the Social Sciences (SPSS) version 28 (2021, Windows version) was used to conduct statistical analysis. Significance was set at p<0.05.

## Results

If the total Fall Risk Assessment Tool score was from five to 11 out of 20 points, then participants were classified as low-risk for falls, from 12 to 15 points, it was moderate-risk, and from 16 points or more, it was high-risk. Within the past three months, there was a total of 51% (n=43) falls in the low-risk category, 40% (n=34) in the moderate-risk, and 8.3% (n=7) in the high-risk.

A total of 87% of participants were on medication, with 25% being males and 62% being females. A total of 7.4% females reported taking one tablet, 10.5% reported taking two tablets and 44% reported taking more than two tablets. In males, 4.7% took one tablet, 3.1% took two, and 17% took more than two. A total of 9.3% of females and 3.1% of males reported not being on any medication.

**Fig. 1:**
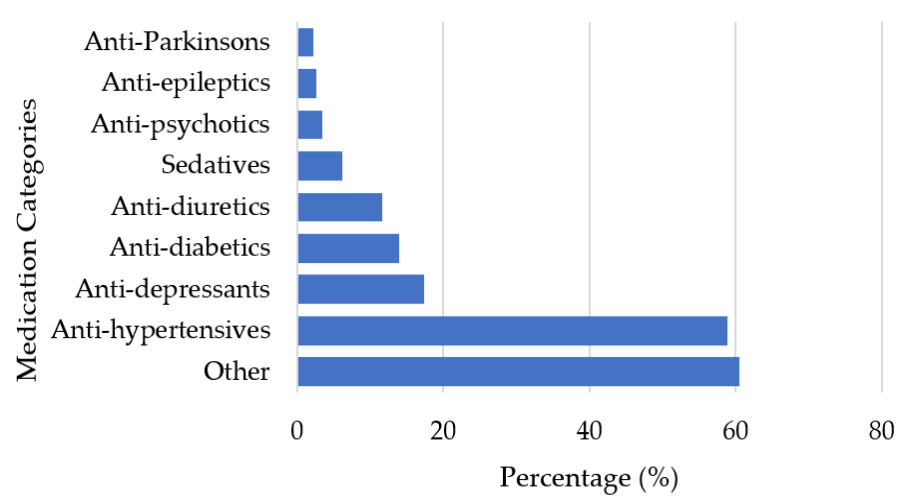
Medications used by the participants (n=258)

The results in Figure 1 illustrate that a total of 461 medications were currently used by the 258 participants of which anti-Parkinson’s (2.3%; n=6), anti-epileptics (2.7%; n=7) and anti-psychotics (3.5%; n=9) made up the smallest percentage of medications used by participants. Sedatives (6.2%; n=16), anti-diuretics (11%; n=30), anti-diabetics (14%; n=36) and antidepressants (17%; n=45) were often used by the participants. Most participants were medicated with anti-hypertensives (58%; n=152) and other medications (60%; n=156) not listed in this study.

In Table 1, the results indicated statistically significant relationships between types of medication and falls, where antidepressants [χ^2^ (1)=4.941; p=0.026, OR=2.08 (95% CI: 0.61, 1.76)] and anti-diabetics [χ^2^ (1)=4.097; p=0.043, OR=2.07 (95% CI: 1.01, 4.22)] were strongly associated with falling. These findings suggest that the use of antidepressant and anti-diabetic medications significantly increases the likelihood of falls. The odds ratio (OR) of 2.08, coupled with a confidence interval (CI) of (1.08, 4.01), indicates that participants taking antidepressants are more than twice as likely to fall. The fact that the entire confidence interval is above 1 (from 1.08 to 4.01) indicates that the association between antidepressants and falls is statistically significant. The odds ratio (OR) of 2.07 for anti-diabetic medication use suggests that these participants are about twice as likely to fall. The confidence interval (1.01, 4.22) indicates a statistically significant relationship, which is slightly higher than the effect of the antidepressant medication.

**Fig. 2:**
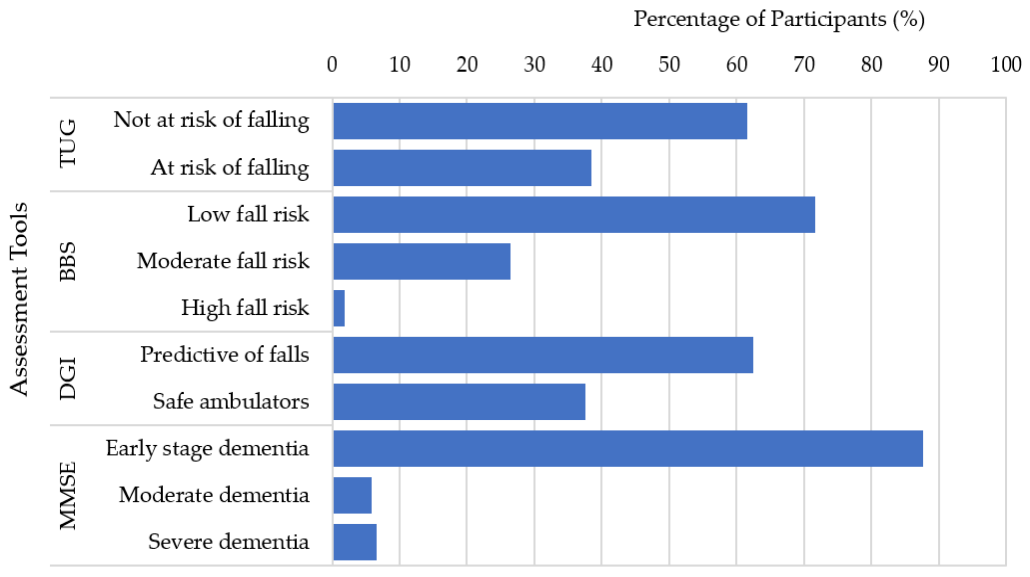
Physical and Cognitive assessment tool outcomes among the participants (n=258). TUG, Timed Up-and-Go; BBS, Berg Balance Scale; DGI, Dynamic Gait Index; MMSE, Mini Mental State Examination

According to the TUG assessment (Figure 2), the majority (61%; n=159) of participants were not at risk of falls, while 38% (n=99) were at risk of falls. Based on the Berg Balance Scale assessment, results indicated that most participants (71%; n=185) had a low risk of falling, while 26% (n=68) had a moderate risk of falling, and 1.9% (n=5) had a high risk of falling. Based on the Dynamic Gait Index assessment, most participants (62%; n=161) were predictive of falling, while only 37% (n=97) had safe ambulation. Furthermore, according to the Mini Mental State Examination, most participants (87%; n=226) were in the early-stage dementia, while 5.8% (n=15) had moderate dementia, and 6.6% (n=17) had severe dementia (Figure 2).

## Discussion

A total of 62% of participants were taking more than two medications, with the majority being female (71%) and being within the age categories of 70–79 years (42%) and 80–89 years (39%). The results indicated that antidepressants and anti-diabetics were the only medications significantly associated with falling. According to two studies, anti-diabetic and anti-psychotic medications were found to be significant determinants of falls, which was comparable to the present study.^[8,9]^ Dhargave and Sendhilkumar (2016), showed multiple medication use that is comparable to the present study.^[10]^ Similar to this study, Kalula et al. reported that the number of medications was also significantly associated with falls.^[11]^

In a study conducted by Richardson et al., multiple medication use was not associated with an increased risk of falls; however, in combination with antidepressant medication use, a greater number of falls and injurious falls was associated with it.^[12]^ These results yielded similar results to the current study, indicating that antidepressant medication was associated with a higher fall risk. The identified falling risks could be attributed to the potential drug interactions between antidepressants and various other medications.^[12]^

Almegbel et al. found that falls among older adults were higher with multiple medication use.^[13]^ This systematic review concluded that the likelihood of falls increased dramatically with the use of four or more medications. According to two retrospective studies, anti-diabetic and anti-psychotic medications were found to be significant determinants of falls, which is comparable to the present study.^[8,9]^ A prospective study suggested that the reduced use of psychotropic medications resulted in a decreased fall rate, which strongly supported the relationship between psychotropic drug use and falling.^[14]^ These results were also consistent with a study among community-dwelling adults in Germany.^[15]^ Even though medication can treat depression, the use of medication, such as antidepressants, sedatives, and hypnotics, was associated with falls among older adults.^[16]^

In South Africa, where healthcare resources may be limited in some areas, implementing effective, targeted interventions to reduce fall risk is crucial. In South Africa, where access to healthcare professionals may be uneven, LTC facilities should consider forming partnerships with local clinics or pharmacies to monitor medication use and identify potentially harmful medications regularly. The implementation of proper healthcare professionals, such as physiotherapists, biokineticists and occupational therapists, should be strongly considered in these facilities. In turn, these multi-disciplinary teams can prioritise communication with staff members and families to educate them on the importance of fall prevention strategies.

According to the physical performance assessments, the results in the present study indicated that the Timed Up-and-Go and Berg Balance Scale were significantly associated with falls. In a systematic review by Park, the Timed Up-and-Go and Berg Balance Scale were used to assess daily activities, such as walking and climbing stairs.^[17]^ The Berg Balance Scale can help healthcare staff assess the risk of falling and prevent or predict falls, especially among older adults.^[18]^ Similarly, the Timed Up-and-Go assessment is a reliable predictor of fall risk and has been recommended as a screening tool in guidelines published by the American Geriatric Society, the British Geriatric Society, and the National Institute for Clinical Excellence (NICE).^[19,20]^ As the number of medications increases, so do the interactions thereof, potentially aggravating the central nervous system effects, which further impair balance and coordination.^[19,12]^ Additionally, complex medication care could compromise adherence and increase the risk of medication errors or even missed doses, which could potentially lead to acute events such as dizziness, hypotension, or hypoglycaemia, which in turn heightens the risk of falls among the elderly. Many studies have indicated that the use of four or more medications is associated with a decline in gait speed, frailty progression, and poor executive function, all of which heighten susceptibility to falling.^[19,20]^ The dynamics of falls are such a concerning matter that screening alone for fall risk not only identifies residents at risk of falling but also enables the identification of areas of concern, such as the risk factors listed in the Fall Risk Assessment Tool.^[21]^

A systematic review found that the incidence of falls among older adults in LTC facilities was 43%, which decreased from 1998 to 202.^[22]^ The results of their study suggested that physical assessments, disease, and medication use should be important factors in assessing fall risk among older adults in LTC facilities.^[22]^ Therefore, when screening residents, it not only protects the individual but also minimises errors and increases the opportunities for healthcare professionals to intervene and provide direct care to the vulnerable.

### Recommendations

Regular review of medication care is crucial, especially for older adults who take multiple medications. Particular attention should be given to medications known to increase fall risk, such as antidepressants and anti-diabetics. In addition to pharmacological considerations, clinicians should incorporate non-pharmacological strategies, including physical therapy, strength training, and balance exercises, to enhance physical function and mitigate fall risk. Structured exercise programs targeting strength and balance have been shown to significantly reduce the likelihood of falls, particularly among older adults managing chronic conditions with medication.

Within long-term care settings, several strategies can be adopted to address these issues. Firstly, LTC facilities should establish formal partnerships with nearby public or private clinics and pharmacies. These partnerships may involve regular on-site visits by nurses, pharmacists, or other healthcare professionals to conduct routine health screenings, review medications, and support the management of chronic diseases.

Secondly, the implementation of multidisciplinary care teams is vital. Collaborations with local universities and health training institutions can facilitate student placements or internships in fields such as nursing, biokinetics, physiotherapy, occupational therapy, and dietetics. These multidisciplinary teams can conduct weekly meetings to review and monitor resident care plans, promoting coordinated care.

Thirdly, staff training should be prioritised to enhance service delivery. LTC management should implement training programs that cover geriatric care, fall prevention, safe mobility practices, and the use of non-pharmacological interventions. These sessions can be delivered by health professionals, supported by the Western Cape Department of Health, to ensure staff are adequately equipped to meet the complex needs of the elderly residents.

### Limitations

Despite the valuable insights this study provides, several limitations should be acknowledged. First, the cross-sectional design of the study limits the ability to establish causal relationships between medication use and falls. A longitudinal design would enable a more robust analysis of how changes in medication over time may influence fall risk among older adults. Second, recall bias in self-reported medication use could have affected the accuracy of the data, as participants may not have accurately remembered or reported their medication use. This could result in either underreporting or overreporting of certain medications. To minimise this bias, future studies could utilise more objective measures of medication use, such as medication records from healthcare providers. Thirdly, selection bias from convenience sampling. As participants were not randomly selected, the sample may not be fully representative of the broader population, thereby limiting the generalisability of the findings. Lastly, there was a lack of control for potential confounding variables such as environmental hazards and varying levels of physical activity among participants. While individuals with severe mobility impairments were excluded, no standardised assessment or control was implemented to account for differences in participants’ physical activity levels or the environmental conditions of their living spaces. These unmeasured confounders may have influenced the outcomes and should be considered when interpreting the findings.

## Conclusion

Falls are common among older adults, and as the proportion of this population continues to increase, falls are expected to pose a significant burden on healthcare systems. One key strategy in fall prevention is the identification and management of modifiable factors, especially medication use. Medications are well-recognised contributors to fall risk; therefore, healthcare providers must be equipped with quality information regarding the fall-related risks of pharmacological treatments. This enables informed decision-making before initiating therapy and supports efforts to reduce the risk of falls among this vulnerable group.

## Figures and Tables

**Table 1:** Association between falling and medication use

Medications	χ2	p-value	Odds ratio	95% CI

Anti-hypertensives	0.019	0.890	1.03	0.611–1.763
Anti-epileptics	0.052	0.819	0.82	0.157–4.340
Anti-psychotics	2.246	0.134	2.69	0.703–10.289
Anti-diuretics	0.537	0.464	0.72	0.309–1.710
Anti-Parkinsons	0.002	0.967	1.03	0.186–5.776
Antidepressants	4.941	0.026*	2.08	1.082–4.012
Sedatives	0.190	0.663	1.26	0.443–3.596
Anti-diabetics	4.097	0.043*	2.07	1.013–4.228
Other	0.003	0.955	1.01	0.596–1.730

* indicates statistically significant association p<0.05 OR (95% CI) indicates odds ratio (95% confidence interval)

## Data Availability

Data sharing will be available on request.
